# Quantitative Model for Efficient Temporal Targeting of Tumor Cells and Neovasculature

**DOI:** 10.1155/2011/790721

**Published:** 2011-03-08

**Authors:** M. Kohandel, C. A. Haselwandter, M. Kardar, S. Sengupta, S. Sivaloganathan

**Affiliations:** ^1^Department of Applied Mathematics, University of Waterloo, Waterloo, ON, Canada N2L 3G1; ^2^Center for Mathematical Medicine, Fields Institute for Research in Mathematical Sciences, Toronto, ON, Canada M5T 3J1; ^3^Department of Physics, Massachusetts Institute of Technology, Cambridge, MA 02139, USA; ^4^Department of Applied Physics, California Institute of Technology, Pasadena, CA 91125, USA; ^5^Department of Medicine, BWH-HST Center for Biomedical Engineering, Brigham and Women's Hospital, Cambridge, MA 02139, USA

## Abstract

The combination of cytotoxic therapies and antiangiogenic agents is emerging as a most promising strategy in the treatment of malignant tumors. However, the timing and sequencing of these treatments seem to play essential roles in achieving a synergic outcome. Using a mathematical modeling approach that is grounded on available experimental data, we investigate the spatial and temporal targeting of tumor cells and neovasculature with a nanoscale delivery system. Our model suggests that the experimental success of the nanoscale delivery system depends crucially on the trapping of chemotherapeutic agents within the tumor tissue. The numerical results also indicate that substantial further improvements in the efficiency of the nanoscale delivery system can be achieved through an adjustment of the temporal targeting mechanism.

## 1. Introduction

The growth of a tumor beyond an avascular state requires the expansion of its vascular network, a process which is realized through the recruitment of host vasculature (angiogenesis) and/or vasculogenesis. Although the inhibition of tumor angiogenesis represents a promising approach to the treatment and control of cancers, recent preclinical studies have suggested that currently available antiangiogenic strategies are unlikely to produce significant therapeutic gains on their own but rather will need to be used in combination with conventional treatments to achieve maximal benefit [1, 2]. To date, however, experimental studies combining antiangiogenic and cytotoxic therapies have shown mixed results [3–9], perhaps in part due to differences in scheduling and sequencing of these modalities. 

Currently, one major challenge to the successful combination of conventional and antiangiogenic therapies is that the administration of an antiangiogenic agent impairs blood flow inside the tumor microenvironment, thus preventing efficient delivery of the chemotherapeutic agent. This difficulty must also be reconciled with the emerging notion of “normalization” of tumor vasculature. The tumor vascular network that arises from abnormal angiogenesis is spatially and temporally heterogeneous with defective endothelium, basement membrane, and pericyte coverage and is characterized by interstitial hypertension, hypoxia, and acidosis [2]. Although high global blood flow is a feature of many tumors, the irregular tumor vasculature is very *inefficient* at delivering nutrients, as well as chemotherapeutic drugs, to malignant cells. It has been suggested [1, 2] that the judicious administration of certain antiangiogenic agents can structurally and functionally “normalize” the abnormal tumor vascular network, rendering the vasculature more conducive to the efficient delivery of both drugs and nutrients to the targeted cancer cells. This transient normalization is characterized by more regular vascular morphology and basement membrane structure, increased pericyte coverage, and decreased hypoxia and interstitial fluid pressure. Recent experimental and clinical studies have shown that blockade of VEGF (vascular endothelial growth factor) signaling, passively prunes some of the immature and leaky vessels of tumors, and actively remodels the remaining vasculature, resulting in a more normalized network [10–12]. Even more recently, it has been shown that creation of perivascular nitric oxide gradients may also result in the normalization of tumor vasculature [13].

A severe limitation to taking advantage of a normalized vascular network is that such a state lasts for only a short period of time [12, 14–16]. After the transient window of vascular normalization has passed, both tumor oxygenation and penetration of chemotherapeutic drugs decrease. The ensuing hypoxia activates hypoxia-inducible factor (HIF-1*α*, upregulating many genes involved in angiogenesis, and renders tumor cells resistant to chemotherapeutic agents [17, 18]. Thus, spatial and temporal tumor targeting plays a critical role in devising efficient combination therapeutic strategies.

**Figure 1:**
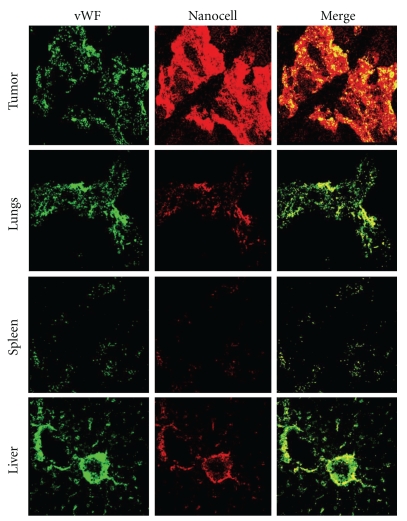
Confocal micrographs of tissue cross sections harvested from tumor-bearing mice at 24 h after injection with imaging-nanocells. Mice were injected with nanocells labeled with semiconductor nanocrystals (quantum dots) to monitor the distribution and leakage from the vessels in the tumor and normal tissues. The sections were immunostained for von Willebrand Factor (vWF) to delineate vasculature. Images were captured using a Zeiss LSM510 confocal microscope at 512 × 512 pixel resolution. The sections were excited with a 488 nm laser, and emission was absorbed at FITC (vWF) and Rhodamine (QD) wavelengths. The nanocells were found to be spatially restricted within normal vasculature as seen in the overlap (yellow) of the red and green signal in the merge images but extravasate out from the tumor vasculature at 24 h as seen by the predominantly red signal (merge).

Sengupta et al. [19] recently designed a novel delivery system (termed a nanocell) comprising a nanoscale pegylated-lipid envelope coating a nuclear nanoparticle. A chemotherapeutic agent (doxorubicin) is conjugated to the nanoparticle and an antiangiogenic agent (combretastatin) is trapped within the lipid envelope. The nanocells extravasate into the tumor through the enhanced permeation and retention (EPR) effect [20, 21] (see Figure 1), a consequence of the highly leaky nature of tumor vasculature (having pores with diameters of 400–600 nm). This is clearly visible in Figure 1 by the preferential accumulation of nanocells (labeled with quantum dots) in the tumor compared to other vascularised tissues: the nanocells are spatially restricted within normal vasculature but extravasate out from the tumor vasculature [19]. The rapid release of the antiangiogenic agent results in at least a partial collapse of the network of tumor blood vessels. The entrapped nanoparticles then slowly release the chemotherapeutic drugs, which remain localized due to the disruption of the nearby vasculature. Sengupta et al. [19] compared the effects of sequential drug delivery using nanocells with several conventional approaches on mice with B16 : F10 melanomas or Lewis lung carcinomas. Animals treated with nanocells containing both drugs showed a better tumor response than any of the other treatment groups. 

While the nanoscale delivery approach outlined above produced a markedly improved effect on tumor control, there remains potential for further refinement of the release kinetics. This motivated us to adapt a mathematical model [22], which incorporates tumor cells, vascular network, as well as their interplay, and the effects of chemotherapy and antiangiogenic therapy to the experimental system studied by Sengupta et al. The details of this model are described in Sections 2 and 3 which discuss the mechanism for the synergistic effect of the nanocell treatment suggested by our model, and how it may be possible to improve the efficiency of the nanocell treatment even further.

## 2. Methods

In order to devise an efficient combination of cytotoxic and antiangiogenic therapies, it is essential to take into consideration the mechanism and timing of tumor vessel response to antiangiogenic agents, as well as the coupling between tumor growth, the vascular network, and response to cytotoxic agents. We have recently developed a mathematical model that incorporates tumor cells and the vascular network, as well as their interactions, and applied it to study the combination of antiangiogenic and radiotherapeutic treatments [22]. The experimental data of  Winkler et al. [12] were used to estimate the model parameters and validate its predictions. The results indicated that application of antiangiogenic therapy, which temporarily results in better delivery of therapeutic agents, in advance of radiotherapy, is the most effective approach, consistent with the experimental results. 

In this paper, we build upon a previous mathematical model [22] by including the effects of chemotherapy and the sequential release kinetics of nanocells. In formulating the model our guiding principle is to make minimal assumptions about the underlying phenomenology of cellular processes while incorporating the essential features of the experiments by Sengupta et al. [19]. A key feature of the latter is the release kinetics of the drugs in nanocells: combretastatin has a rapid release (reaching significant levels within 12 h), while doxorubicin releases more slowly extending over 15 days (compared to approximately 4 days for liposomes). Our mathematical model incorporates this temporal targeting profile and allows complete control over other possible factors contributing to the increased effectiveness of the nanocell treatment, such as differences in the total amount of drugs delivered to the cancer cells. Thus, we can directly test and further investigate the temporal targeting mechanism proposed by Sengupta et al. [19].

### 2.1. Tumor Cells and Vascular Network

Following previous studies of tumor growth, we model the density of cancer cells (at position *x* at time *t*) by a spatiotemporal field *n*(*x*, *t*) according to a variant of the Fisher equation (below). The novel aspect of our approach is to similarly introduce a field *m*(*x*, *t*) to model the density of blood vessels. This circumvents accounting for the precise nature of blood flow and, as demonstrated earlier [22], the key features of the interplay between growth and blood supply can be captured by the evolution equations:

(1) $$\begin{array}{c} \begin{array}{c} \frac{\partial n}{\partial t}=\nabla^{2}n+n\left(1-n\right)+{\tilde{\alpha }}_{1}m\left(x,t\right)n,\; \end{array} \end{array}$$

(2) $$\begin{array}{c} \frac{\partial m}{\partial t}={\tilde{D}}_{2}\nabla^{2}m+m\left[\tilde{\alpha }+\tilde{\beta }m+\tilde{\gamma }m^{2}\right]+{\tilde{\alpha }}_{2}n\left(x,t\right)m. \end{array}$$

For simplicity, the above equations are presented in dimensionless form. They are related to the corresponding dimensionful equations via the transformations *t* → *ρt*, $x\to \sqrt{\rho /D_{1}}x$, and *n* → *n*/*n*_lim _ [22], where *ρ* is the net proliferation rate, *D*_1_ is the diffusion coefficient of tumor cells, and *n*_lim _ is the “carrying capacity” for tumor cells.

For ${\tilde{\alpha }}_{1}=0$, (1) is the (dimensionless) Fisher equation, which has two fixed points: an unstable fixed point at n* = 0 (no population at all) and a stable fixed point at n* = 1 (where the population saturates to the carrying capacity). In the absence of the nonlinear term, that is, for the simple exponential form and ${\tilde{\alpha }}_{1}=0$, integrating both sides of (1) leads, for a constant diffusion coefficient, to a simple exponential increase in the number of cells. The growth of a tumor beyond an avascular state (up to a maximum size of about 1-2 mm in diameter) requires the development of a vascular network. The additional term ${\tilde{\alpha }}_{1}mn$ in (1) indicates that tumor growth is enhanced by the presence of vasculature. Mathematically, this results in a stable fixed point at $n^{*}=1+{\tilde{\alpha }}_{1}m^{*}$; see the following. 

Following Kohandel et al. [22] (equations (6) and (8) of Kohandel et al. [22] contain typos in the interaction terms *α*_1_ *K*(*x*, *t*)*c* and ${\tilde{\alpha }}_{1}K(x,t)c$ which, in the notation of Kohandel et al. [22], should in fact read *α*_1_ *m*(*x*, *t*)*c* and ${\tilde{\alpha }}_{1}m(x,t)c$, resp.), we use (2) to take into account the heterogeneous tumor vasculature. This coarse-grained model, instead of the exact pattern of vessels, produces islands of vascular and nonvascular networks. For ${\tilde{\alpha }}_{2}=0$, and setting $\tilde{\alpha }=-1$, $\tilde{\beta }=3$, and $\tilde{\gamma }=-2$, we obtain two stable fixed points for m* at 0 and 1 and an unstable one at 1/2. Starting from a random (positively distributed and close to zero) initial configuration for *m*(*x*, 0), (2) produces randomly distributed islands of *m* = 1 (vascular) and *m* = 0 (non vascular). The last term in (2), ${\tilde{\alpha }}_{2}nm$, represents the effect of tumor cells on the development of vessels. We assume that the tumor cells produce the proangiogenic cytokines, leading to the extension of the vascular network; in our phenomenological approach, we assume that the higher density of cancer cells creates higher vascular density. In fact, a nonzero ${\tilde{\alpha }}_{2}$ shifts the stable fixed points to

(3) $$m^{*}=\frac{1}{4}\left(3+{\tilde{\alpha }}_{1}{\tilde{\alpha }}_{2}\pm \sqrt{8\left({\tilde{\alpha }}_{2}-1\right)+{\left(3+{\tilde{\alpha }}_{1}{\tilde{\alpha }}_{2}\right)}^{2}}\right).$$

For example, for ${\tilde{\alpha }}_{1}=1.1$ and ${\tilde{\alpha }}_{2}=0.9$ (obtained from fits to experimental data, see the results and discussion section), m* = 0.02, 1.97. Thus the fixed point at 1 moves to a higher value, indicating that the tumor vascular density is higher than in the corresponding normal tissue. This increased value will also be utilized in modeling of the poor delivery characteristics of the tumor vasculature, as well as the effect of antiangiogenic therapy; see below.

### 2.2. Delivery of Nanocells and Liposomes

Following the same strategy as above, we model the spatiotemporal variations of liposomes and nanocells by concentration fields *C*_*i*_(*x*, *t*), the discrete index *i* labeling the drug administration at time *t*_*i*_. The permeation of nanoparticles within a tumor depends on their sizes; large nanoparticles of the order of 100 nm (which is the case in Sengupta et al. 2007 experiments) appear to stay close to the vasculature [23]. Hence, we assume that diffusion of liposomes and nanocells within the tissue surrounding the tumor, as well as reabsorption of these particles into the blood vessels, can be neglected. The evolution of the field *C*_*i*_(*x*, *t*) is then modeled by the dimensionless equation,

(4) $$\frac{\partial C_{i}}{\partial t}=\tilde{\delta }\Gamma_{i}\left(t\right)m\left(x,t\right)\exp\;\;\left[-{\left(\frac{m\left(x,t\right)}{m_{\lim\;}}\right)}^{2}\right].$$

The function Γ_*i*_(*t*) represents the (average) concentration of the liposomes and nanocells in the blood vessels (see Section 2.4). The tumor vasculature is structurally and functionally abnormal, and the vessels are very inefficient at delivering nutrients and chemotherapeutic drugs. This poor delivery could be due to defective vascular structure, lack of perfusion of tumor blood vessels, inconsistent flow, and elevated interstitial fluid pressure [24]. However, there is growing evidence that vascular efficiency can be improved with antiangiogenic therapy through the “normalization” process [1, 2]. In (4), the poor delivery of tumor vessels is modeled by the function *m* exp [−(*m*/*m*_lim _)^2^]. For $m_{\lim\;}=\sqrt{2}$, this function has a maximum at *m* = 1, corresponding to the efficient delivery of normal vessels. For *m* > 1, which corresponds to tumor vasculature (e.g., ${\tilde{\alpha }}_{1}=1.1$ and ${\tilde{\alpha }}_{2}=0.9$ gives m* = 1.97, as mentioned in the previous section), the delivery decreases. Finally, *m* < 1 corresponds to immature or degraded vessels, resulting again in inefficient nutrient or drug delivery. Hence, for a tumor vasculature, decreasing the field *m* to values close to one, by the administration of an antiangiogenic agent, results in improved vascular efficiency and better delivery of nutrients and chemotherapeutic agents [22]. The exponential term therefore accounts for the poor delivery of vasculature as well as the increase in the delivery of liposomes and nanocells to tumor cells through normalization. One should note that strong dosage of the antiangiogenic drug may lead to values of *m* less than one, leading to either poor delivery through immature vessels or complete regression of the vasculature [24].

### 2.3. Drug Release from Nanocells and Liposomes

Next, we denote by *c*(*x*, *t*) and *d*(*x*, *t*) the concentrations of free antiangiogenic and chemotherapeutic agents released from liposomes and nanocells into the tumor tissue, respectively. The temporal and spatial evolution of these fields is modeled by the dimensionless equations

(5) $$\begin{array}{c} \frac{\partial c}{\partial t}={\tilde{D}}_{3}\nabla^{2}c+{\tilde{\lambda }}^{\left(C\right)}R_{j}^{\left(C\right)}\left(x,t\right)-{\tilde{\nu }}^{\left(C\right)}c, \end{array}$$

(6) $$\begin{array}{c} \frac{\partial d}{\partial t}={\tilde{D}}_{4}\nabla^{2}d+{\tilde{\lambda }}^{\left(D\right)}R_{j}^{\left(D\right)}\left(x,t\right)-\tilde{\mu }m\left(x,t\right)d-{\tilde{\nu }}^{\left(D\right)}d. \end{array}$$

We shall contrast the four types of treatment tested in the experiments via the index *j* = 1–4, denoting chemotherapy (NC[D], *j* = 1), antiangiogenic therapy (L[C], *j* = 2), simple liposome encapsulating both (L[CD], *j* = 3), and nanocells (NC[CD], *j* = 4). Free antiangiogenesis and chemotherapy agents released from nanocells and liposomes are small enough to diffuse through the tumor tissue (first terms in the right side of (5) and (6); ${\tilde{D}}_{3}$ and ${\tilde{D}}_{4}$ are dimensionless diffusion coefficients). The term $-\tilde{\mu }m(x,t)d$ describes the reabsorption of free chemotherapy drugs into the blood vessels. We assume that no such term is present in the equation for *c*(*x*, *t*) since antiangiogenic drugs act on normal as well as on abnormal blood vessels, which prevents absorption. However, both equations involve terms of the form $-{\tilde{\nu }}^{\left(C\right)}c$ and $-{\tilde{\nu }}^{\left(D\right)}d$, which describe the natural decay of free drugs. The release of free chemotherapy and antiangiogenesis agents in (5) and (6), that is, ${\tilde{\lambda }}^{\left(C,D\right)}R_{j}^{\left(C,D\right)}$, proceeds according to

(7) $$R_{j}^{\left(C,D\right)}\left(x,t\right)=\sum_{i}\text{R}{\text{P}}_{i,j}^{\left(C,D\right)}\left(t\right)C_{i}\left(x,t\right),$$

in which the sum runs over all administration times *t*_*i*_. The release profiles RP_*i*,*j*_^(*C*,*D*)^(*t*) are either identical to zero or satisfy

(8) $$\sum_{i}\overset{t_{f}}{\underset{0}{\int }}\text{R}{\text{P}}_{i,j}^{\left(C,D\right)}\left(t\right)dt=1,$$

where, as above, the sum runs over all administration times and *t*_*f*_ = 17 days is the longest time considered in the experiments by Sengupta et al. [19]. The above condition on the release profiles fixes the total amount of drugs released from liposomes and nanocells over the time interval considered in the experiments by Sengupta et al. [19] and, thus, ensures a fair comparison of different therapeutic strategies in our model. 

Finally, the effects of chemotherapy and antiangiogenic therapy on cancer cells and blood vessels are modeled by

(9) $$\begin{array}{c} {\frac{\partial n\left(x,t\right)}{\partial t}|}_{\text{chemo}}=-{\tilde{A}}^{\left(D\right)}d\left(x,t\right)n,\;\text{for}\;\;j=1,3,4,\\ {\frac{\partial m\left(x,t\right)}{\partial t}|}_{\text{anti}}=-{\tilde{A}}^{\left(C\right)}c\left(x,t\right)m,\;\text{for}\;\;j=2,3,4, \end{array}$$

where the vertical lines indicate that the above terms are added to (1) and (2), respectively. Here, ${\tilde{A}}^{\left(D\right)}$ and ${\tilde{A}}^{\left(C\right)}$ represent the strength of chemotherapy and antiangiogenic therapy, respectively; *d*(*x*, *t*) and *c*(*x*, *t*) are defined by (5) and (6). The details of the above model and its parameterization are discussed in the following.

### 2.4. Release Profiles

To determine the release profiles, RP_*i*,*j*_^(*C*,*D*)^(*t*) for *j* = 1–4, we first note that NC[D] only involves chemotherapy (but does not contain antiangiogenic therapy); thus, RP_*i*,1_^(*C*)^(*t*) = 0; recall that NC[D] is denoted by *j* = 1. Similarly, RP_*i*,2_^(*D*)^(*t*) = 0 since L[C] (*j* = 2) only involves antiangiogenic therapy. The remaining release profiles should ideally be fixed from* in vivo* release experiments. We expect that the release profiles of combretastatin and doxorubicin in L[CD] are similar to the release profile of combretastatin in L[C] that is, RP_*i*,3_^(*C*)^(*t*) = RP_*i*,3_^(*D*)^(*t*) = RP_*i*,2_^(*C*)^(*t*)—this is due to the fact that for all these cases, drugs are included inside a liposome. Similarly, since all nanocells (independently of whether they contain combretastatin or not) have liposome on the outer layer, we have RP_*i*,1_^(*D*)^(*t*) = RP_*i*,4_^(*D*)^(*t*). Thus, we need to determine the functions RP_*i*,1_^(*D*)^(*t*), RP_*i*,2_^(*C*)^(*t*), and RP_*i*,4_^(*C*)^(*t*). The *in vitro* studies by Sengupta et al. [19] show that the release of doxorubicin from NC[D] is delayed relative to the release of combretastatin from the liposome, with an extending time of approximately 4 days for the liposome and an extending time of approximately 15 days for the core of the nanocell. On that basis we take

(10) $$\begin{array}{c} \text{R}{\text{P}}_{i,1}^{\left(D\right)}\left(t\right)=\frac{1}{N_{1}}\theta \left(t-t_{i}\right){\left(t-t_{i}\right)}^{p_{\text{NC}}}\exp\;\;\left(-\frac{\left(t-t_{i}\right)}{\tau_{\text{NC}}}\right),\\ \text{R}{\text{P}}_{i,2}^{\left(C\right)}\left(t\right)=\frac{1}{N_{2}}\theta \left(t-t_{i}\right){\left(t-t_{i}\right)}^{p_{L}}\exp\;\;\left(-\frac{\left(t-t_{i}\right)}{\tau_{L}}\right),\\ \text{R}{\text{P}}_{i,4}^{\left(C\right)}\left(t\right)=\frac{1}{N_{4}}\theta \left(t-t_{i}\right){\left(t-t_{i}\right)}^{p_{L}^{\prime }}\exp\;\;\left(-\frac{\left(t-t_{i}\right)}{\tau_{L}^{\prime }}\right), \end{array}$$

where *t*_*i*_ = 8,10,12,14,16 days are the administration times used in the experiments by Sengupta et al. [19], the constants *N*_1,2,4_ are determined from the normalization condition, see (7), and *θ*(*x*) is the unit step function (defined by *θ*(*x*) = 1 for *x* ≥ 1 and *θ*(*x*) = 0 otherwise). Assuming that the release profile of L[C] and the liposome coating of the nanocell are similar, we can set *p*_*L*_ = *p*_*L*_′ and *τ*_*L*_ = *τ*_*L*_′, in which case (7) implies *N*_2_ = *N*_4_. Based on Sengupta et al. [19] we take *p*_NC_ = 0.3 and *τ*_NC_ = 15 days, and *p*_*L*_ = 0.1 and *τ*_*L*_ = 2 days. For our modified nanocell therapy (see Section 3) we use the same treatment schedules as for NC[CD] but take *p*_NC_ = 0.8 in (10) with an appropriate normalization factor determined from (7). This ensures that for all combined therapies the nanocells and liposomes release the same total amount of combretastatin and doxorubicin.

The function Γ_*i*_(*t*) in (4) describes the (average) concentration of the liposomes and nanocells in the blood vessels. In using the same function Γ_*i*_(*t*) for liposomes and nanocells we assume that, once a therapy has been administered, changes to the drug concentration in the blood vessels only result from some natural decay (e.g., adsorption) independent of the chemical composition of the particles. Thus, we set

(11) $$\Gamma_{i}\left(t\right)=\theta \left(t-t_{i}\right)\exp\;\;\left(-\frac{\left(t-t_{i}\right)}{\tau_{D}}\right),$$

where, as above, *t*_*i*_ are again the administration times. There is some indication (Sengupta et al. [19], see also Figure 1) that Γ_*i*_(*t*) is different for liposomes and nanocells, with an increased delivery of nanocells into the tissue surrounding the tumor through a mechanism other than vascular normalization. This would further increase the effect of NC[CD] relative to L[CD] through an increase in the amount of drugs delivered to the tumor. However, in order to allow a direct investigation of the temporal targeting mechanism proposed by Sengupta et al. [19], we take the Γ_*i*_(*t*) for conventional therapies to be the same as for the nanocell treatment and set *τ*_*D*_ = *τ*_NC_.

### 2.5. Parameterization

Free antiangiogenesis and chemotherapy agents are small particles which can be assumed to have diffusion characteristics similar to nutrients such as oxygen, for which experimental data is readily available. Thus, we set ${\tilde{D}}_{3}={\tilde{D}}_{4}$ in (5) and (6) and use a value of ${\tilde{D}}_{3}$ similar to the diffusion constant of free oxygen in Kohandel et al. [22]. As mentioned earlier, the field *m*(*x*, *t*) stands for the average distribution of blood vessels (rather than the exact pattern of vasculature); thus, the diffusion coefficients for drugs (or nutrients) are not of the same order as diffusion from a single blood vessel. Moreover, because chemotherapy agents released from the nanocells are transported away through blood vessels in the same way for NC[D] and NC[CD], and ${\tilde{D}}_{3}={\tilde{D}}_{4}$, we use the same reabsorption rate $\tilde{\mu }$ for all treatments. Similarly as in Kohandel et al. [22] for free oxygen, we assume that the natural decay of *c* and *d* is not too strong and, hence, that the decay constants ${\tilde{\nu }}^{\left(C\right)}$ and ${\tilde{\nu }}^{\left(D\right)}$ take smaller numerical values than $\tilde{\mu }$. Furthermore, combretastatin released from the liposome decays faster than doxorubicin released from the nanocells [19], and, thus, we take ${\tilde{\nu }}^{\left(C\right)}>{\tilde{\nu }}^{\left(D\right)}$. On the other hand, one may expect that doxorubicin released from the liposome during treatment with L[CD] decays faster than doxorubicin released from the nanocells. This would further decrease the efficiency of L[CD] relative to NC[CD], but to avoid a proliferation of parameters we use the same value ${\tilde{\nu }}^{\left(D\right)}$ for all treatments. 

The amount of drugs administered can be included in our model through the values of the coefficients ${\tilde{\lambda }}^{\left(C\right)}$ and ${\tilde{\lambda }}^{\left(D\right)}$. In the experiments described by Sengupta et al. [19] approximately one hundred times more combretastatin than doxorubicin was injected, and, hence, we take ${\tilde{\lambda }}^{\left(C\right)}=100\;{\tilde{\lambda }}^{\left(D\right)}$. Moreover, the constants ${\tilde{A}}^{\left(C\right)}$ and ${\tilde{A}}^{\left(D\right)}$ determine the effectiveness of a given therapy and are therefore crucial parameters in our model. To allow a quantitative comparison between the effects of different combined therapies, we use the same values ${\tilde{A}}^{\left(C\right)}$ and ${\tilde{A}}^{\left(D\right)}$ for single and combined therapies. It is thereby assumed that the effect of a given therapeutic agent is not influenced by the presence or absence of another therapeutic agent. The values of all parameters appearing in our model are fixed by fitting volume curves for V, L[C], and NC[D] to the corresponding experimental results [19].  Table 1 summarizes the parameter values used in our simulations (see the appendix for the sensitivity analysis of parameters). The results of the combined treatments are then predicted by our model without any further assumptions.

While we consider a variety of different interactions in our model, with the aim of not excluding any possible mechanism for the success of the nanocell treatment *a priori*, we find that only the three parameters *D*_1_, ${\tilde{A}}^{\left(C\right)}$, and ${\tilde{A}}^{\left(D\right)}$ need to be adjusted to distinguish between lung carcinoma and melanoma. Moreover, we also find that for both lung carcinoma and melanoma the success of the nanocell treatment relies crucially on the temporal release profiles used by Sengupta et al. [19] and on the possibility of reabsorption of chemotherapeutic drugs into the bloodstream (see Section 3), which is parameterized through $\tilde{\mu }$. As in Kohandel et al. [22], we use a normalized Gaussian initial condition for *n*(*x*, *t*) with variance *σ* = 0.35 and a random initial condition for *m*(*x*, *t*) evenly distributed between zero and one. Zero initial conditions are considered for the concentrations of the drugs. All simulations are performed on a cubic grid with 50 × 50 × 50 points and no-flux boundary conditions.

## 3. Results and Discussion

### 3.1. Consistency of Model Results

**Figure 2:**
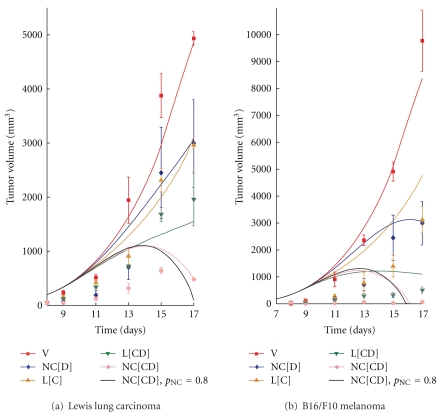
Curves for the tumor volume of (a) lung cancer and (b) melanoma obtained with no treatment (V), nanocells containing only doxorubicin (NC[D]), liposomes containing only combretastatin (L[C]), liposomes with combretastatin and doxorubicin (L[CD]), nanocells with combretastatin and doxorubicin (NC[CD]) and nanocells with combretastatin and doxorubicin but with a delayed release of doxorubicin (NC[CD] and *p*_NC_ = 0.8). The solid curves are obtained by integrating (1)–(6) in Section 2, and the data points are taken from the experiments by Sengupta et al. [19]. The same total amount of drugs is released by liposomes and nanocells for the combined therapeutic strategies, which corresponds to double the amount released for NC[D] and L[C] individually.

To confirm our model, numerical simulations are performed according to the experimental protocol of Sengupta et al. [19] on lung cancer and melanoma (see Figure 2). In these experiments, 2.5 × 10^5^ Lewis lung carcinoma cells or 3 × 10^5^ GFP-positive BL6/F10 melanoma cells were implanted in male C57/BL6 mice, and treatments started when tumors reached 50 mm^3^ in volume (after about 8 days). The kinetics of tumor growth and blood vessel formation, as well as the data points for the control group (V, red), are used to estimate the related model parameters for the case of lung cancer (see Table 1). The experimental treatment schedules, as well as pharmacokinetics and pharmacodynamics of agents, are used in the simulations to fit the data for single administration of antiangiogenic therapy (combretastatin-encapsulated liposomes, L[C], brown) and chemotherapy (nanocells containing doxorubicin but lacking combretastatin, NC[D], blue). The corresponding curves for melanoma are obtained by modifying three model parameters describing the diffusion of cancer cells and the effect of therapeutic agents on the vascular network and cancer cells (see Section 2). We then perform numerical simulations with the estimated parameters to predict the results for the conventional (a liposome encapsulating both doxorubicin and combretastatin, L[CD], green) and nanocell (NC[CD], purple) approaches for the combination of antiangiogenic and cytotoxic treatments. 

As shown in Figure 2, the numerical results (solid lines) obtained from our phenomenological model reproduce the major trends observed in the experiments by Sengupta et al. [19] (points) for lung cancer and melanoma. While the conventional combination of combretastatin and doxorubicin only produces a doubling effect compared to the single administration of combretastatin or doxorubicin, nanocells clearly show a more pronounced effect with the same amount of drugs administered. Our simulations strongly support the hypothesis that the increased effect of the nanocell treatment is mainly due to the temporal release achieved through nanocells [19], which is from a mathematical perspective the only feature distinguishing L[CD] and NC[CD] in our model (see Section 2). This is discussed further in the following.

In the study by Sengupta et al. [19], the results of the single therapies NC[D] and L[C] were also compared to the coadministration of NC[D] and L[C] (NC[D] + L[C]). However, NC[D] + L[C] only showed a negligible effect when compared to either of the treatments alone. This is probably due to a limitation on the total number of nanocells or liposomes which can be taken up by the tumor tissue at any given time. This limitation does not affect any of the other more effective combination strategies considered in Sengupta et al. [19] and, thus, we do not discuss this case in our modeling approach. 

### 3.2. Synergistic Effect of Nanocell Treatment

**Figure 3:**
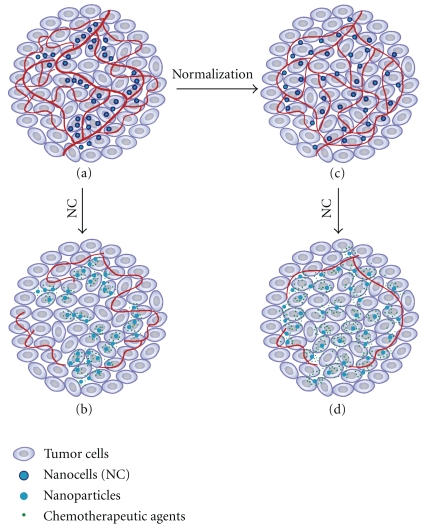
Mechanisms for the temporal targeting of tumor cells and neovasculature. (a) Nanocells are delivered to the tumor tissue through the neovasculature and rapidly release antiangiogenic agents. (b) The vascular collapse leads to the trapping of the chemotherapeutic agents within the tumor tissue and thereby prevents reabsorption into the bloodstream. According to our mathematical model this is the principal mechanism responsible for the superior results of the nanocell treatment found by Sengupta et al. [19]. (c) The normalization of tumor blood vessels produced by the antiangiogenic therapy leads to a transient “window of opportunity” [2] during which the delivery of nanocells into the tumor tissue is enhanced. (d) Our model suggests that through a judicious timing of the release profiles the interplay between normalization and vascular collapse can be utilized to improve the efficacy of the nanocell treatment.

Our model predicts the nanocell treatment can produce a combined effect that is *greater than the sum of its parts *through the two different mechanisms illustrated in Figure 3. First, the transient normalization resulting from antiangiogenic therapy enhances the delivery of additional nanocells to the tumor tissue. This in turn increases the effect of the subsequent release of chemotherapy. Second, antiangiogenic therapy can also cut off the blood supply to the tumor, which effectively traps the cytotoxic drugs within the tumor tissue. Thus, smaller amounts of free chemotherapy agents are transported away from the tumor tissue. According to our simulations, the latter mechanism is crucial for obtaining the superior results of NC[CD] reported by Sengupta et al. [19], while normalization only brings a small increase in the effectiveness of NC[CD] relative to L[CD]. However, as discussed later, normalization can still be employed to further improve the efficacy of the nanocell treatment.

As mentioned earlier, our modelling hypothesis is that by adjusting the interplay between normalization and the temporal targeting described by Sengupta et al. [19] an improved therapeutic outcome can be achieved. To validate this hypothesis, we modeled different release profiles for the nanocell core which delay the secretion of chemotherapy relative to antiangiogenic therapy even further than the release profile considered in the experiments of Sengupta et al. [19] (see Section 3). We were careful to adjust the delayed release profiles such that over the course of the treatment the same total amount of antiangiogenic and chemotherapeutic agents was released as in the combination therapies, L[CD] and NC[CD], considered before. Thus, compared to the conventional nanocell therapy, with our modified release profiles more chemotherapeutic agents are released by nanocells administered at early times, but correspondingly fewer chemotherapeutic drugs are released by nanocells administered at later times.

The simulation results indicate that with the adjusted release kinetics a substantial improvement in the efficacy of the nanocell treatment can be achieved (see the black curves in Figure 2). As illustrated in Figure 3, this improved result is due to a combination of normalization, which is mainly effective in the early stages of the therapy and increases the fraction of nanocells delivered to the tumor tissue at early times, and the vascular shutdown induced by antiangiogenic therapy, which decreases the fraction of nanocells delivered at later times and traps the chemotherapeutic agents within the tumor tissue. Our modified release profiles take advantage of these two effects and lead to better coordination between the arrival of nanocells in the tumor tissue and the release of antiangiogenic and chemotherapeutic agents. Thus, our simulations suggest that, by means of a judicious timing of normalization and the trapping of chemotherapeutic agents in the tumor tissue through the release of combretastatin, a better therapeutic outcome can be achieved.

In summary, mathematical models can be used to simulate various therapeutic scenarios and aid in hypothesis testing “in silico” and conversely to guide experimental research. Following the very successful experimental results of Sengupta et al. [19] and experimental and clinical studies on the normalization process of tumor vasculature [10–12], we have developed a mathematical formulation that captures the qualitative picture proposed by Sengupta and coworkers. On the basis of this model we find that the dramatically improved therapeutic effect of the nanocell treatment demonstrated by Sengupta et al. [19] primarily results from the trapping of chemotherapeutic agents, rather than an increase in the number of nanocells delivered to the tumor through normalization. Moreover, we find that through an adjustment of the release kinetics for chemotherapy it may be possible to substantially improve the efficacy of the nanocell treatment. As a result of our promising computational results, it seems clear that more experimental and preclinical data are required to further validate this strategy for improved therapeutic outcome. In particular, our model results suggest that nanocells with a longer delay in the secretion of chemotherapy relative to antiangiogenic therapy (compared to the release profile considered in the experiments of Sengupta et al. [19]) may further increase the efficiency of the treatment. Our computational approach could be also used to design nanocells for other types of cancers, and to quantitatively determine promising release profiles.

## Figures and Tables

**Figure 4:**
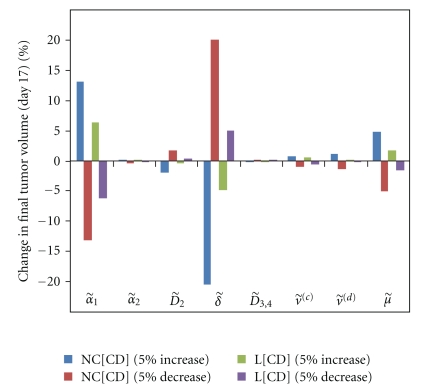
Sensitivity analysis for L[CD] and NC[CD] in Lewis lung carcinoma. Each set comprising four color-coded columns corresponds to the parameter indicated directly below the *x*-axis.

**Figure 5:**
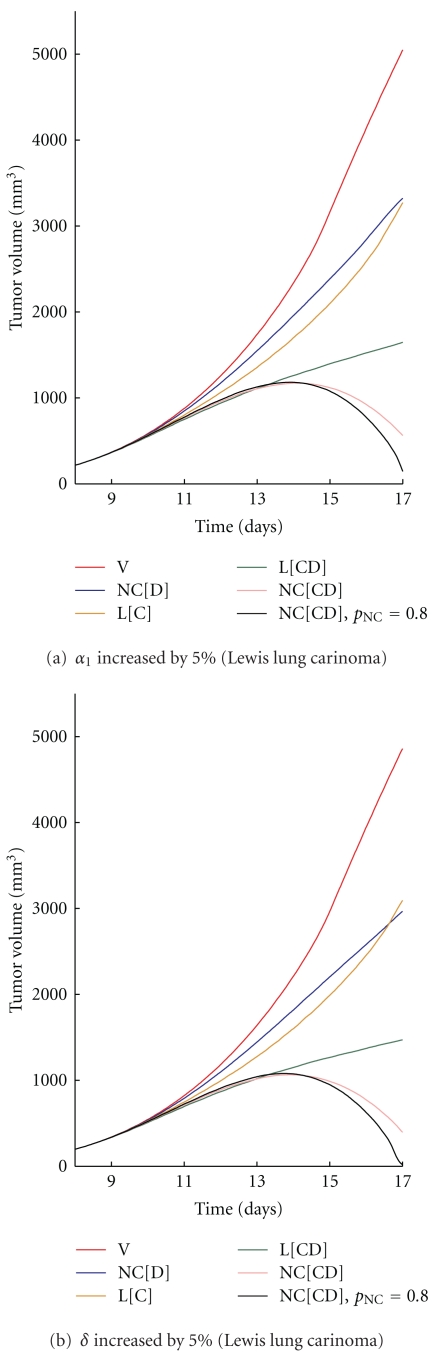
The tumor volume obtained with a 5% increase in (a) ${\tilde{\alpha }}_{1}$ and (b) $\tilde{\delta }$ for Lewis lung carcinoma.

**Table 1:** Numerical values for the parameters in (1) and (9) used in our simulations. In addition to the values shown, we use in the dimensionful equations *D*_1_ = 0.32 mm^2^/day for lung cancer and *D*_1_ = 0.46 mm^2^/day for melanoma, *ρ* = 0.35 day^−1^ for both melanoma and lung cancer (see Section 3.1), and a threshold for the detectability of tumor cells *c*_*th*_ = 0.09 [22]. As explained in the main text we have ${\tilde{D}}_{3}={\tilde{D}}_{4}$ and ${\tilde{\lambda }}^{\left(C\right)}=100{\tilde{\lambda }}^{\left(D\right)}$. The effectiveness of chemotherapy and antiangiogenic therapy are parameterized by ${\tilde{A}}^{\left(D\right)}=1.65$ and ${\tilde{A}}^{\left(C\right)}=0.1$ for lung cancer, and by ${\tilde{A}}^{\left(D\right)}=3$ and ${\tilde{A}}^{\left(C\right)}=0.3$ for melanoma (see Section 3.1).

${\tilde{\alpha }}_{1}$	${\tilde{D}}_{2}$	$\tilde{\alpha }$	$\tilde{\beta }$	$\tilde{\gamma }$	${\tilde{\alpha }}_{2}$	$\tilde{\delta }$	*m*_lim _	${\tilde{D}}_{3}$	${\tilde{\lambda }}^{\left(C\right)}$	${\tilde{\nu }}^{\left(C\right)}$	${\tilde{\nu }}^{\left(D\right)}$	$\tilde{\mu }$
1.1	0.005	–1	3	–2	0.9	0.8	$\sqrt{2}$	0.02	1300	3	0.1	8

## Appendix

In this appendix we investigate the effect of variations in the values of the model parameters on our model predictions. To study parameter sensitivity, we calculate the effect of small changes of the parameters on final tumor volume (at day 17). We determine the percent change in the final tumor volume when the model parameters are perturbed from their estimated values by 5% [25].  Figure 4 shows the results for the case of combination therapies using nanocells and liposomes, that is, NC[CD], and L[CD], in lung cancer. One should note that changing ${\tilde{\lambda }}^{\left(C\right)}=100{\tilde{\lambda }}^{\left(D\right)}$ has the same effect as varying $\tilde{\delta }$; in addition, the parameters $\tilde{\alpha }$, $\tilde{\beta }$, and $\tilde{\gamma }$ are not changed as they are responsible for the creation of the vascular network (see main text). The results indicate that the parameters ${\tilde{\alpha }}_{1}$ and $\tilde{\delta }$ contribute most significantly to the final tumor volume; however, these contributions are smaller in L[CD] compared to NC[CD].

To investigate whether the trends obtained from our model for different treatments are robust with respect to changes in the model parameters, we performed numerical simulations of our model using parameterizations for which ${\tilde{\alpha }}_{1}$ or $\tilde{\delta }$ were increased by 5% while all other model parameters were held fixed. As shown in Figure 5, these variations do not affect the trends illustrated in Figure 2(a).
